# Supplementary material for: Recently activated CD4 T cells in tuberculosis express OX40 as a target for host-directed immunotherapy
Source: Nat Commun. 2023 Dec 19;14:8423. doi: 10.1038/s41467-023-44152-8 (PMC10728168; doi:10.1038/s41467-023-44152-8)
Supplement: Supplementary file 1 — Supplementary Information [file 41467_2023_44152_MOESM1_ESM.pdf]

Supplementary Information

Title: Recently activated CD4 T cells in tuberculosis express OX40 as a target for host-directed immunotherapy

First author: Abigail R. Gress

Corresponding author: Tyler D. Bold

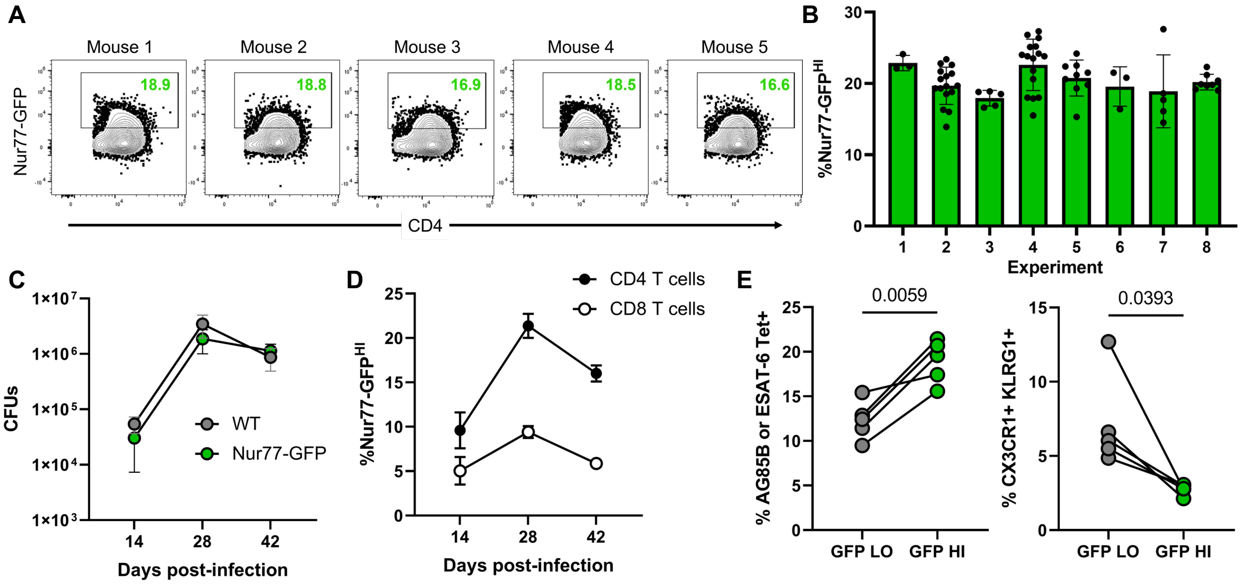

Supplementary figure S1. Nur77-GFP model validation.
(A) Flow cytometry of Nur77-GFP expression among CD4 T cells from 5 Nur77-GFP mice harvested at four weeks post-infection in a single experiment. Gated on $CD3^{+}CD4^{+}CD44^{+}$ cells. **(B)** Frequency of Nur77-GFP^{HI} CD4 T cells from Nur77-GFP mice harvested at four weeks post-infection in multiple experiments. Mice per group: 3, 16, 5, 16, 9, 3, 5, and 8 for experiments 1-8, respectively. Mice per group: 5 for isotype and 10 for OX40 at day 13, 5 each for isotype and OX40 at day 42, and 6 each for isotype and OX40 at day. **(C-D)** Wild type and Nur77-GFP mice harvested at 14, 28, and 42 days post-infection for lung bacterial load and flow cytometry. 3 mice per group. **(C)** Lung bacterial load or **(D)** frequency Nur77-GFP^{HI} CD4 T cells. **(E)** Frequency of Ag85B or ESAT-6 $tetramer^{+}$ and $CX3CR1^{+}KLRG1^{+}$ phenotypes in Nur77-GFP^{LO} and Nur77-GFP^{HI} CD4 T cells harvested from Nur77-GFP mice at four weeks post-infection. P-values calculated with paired two tailed t-test, 5 mice per group. Error bars indicate standard deviation.

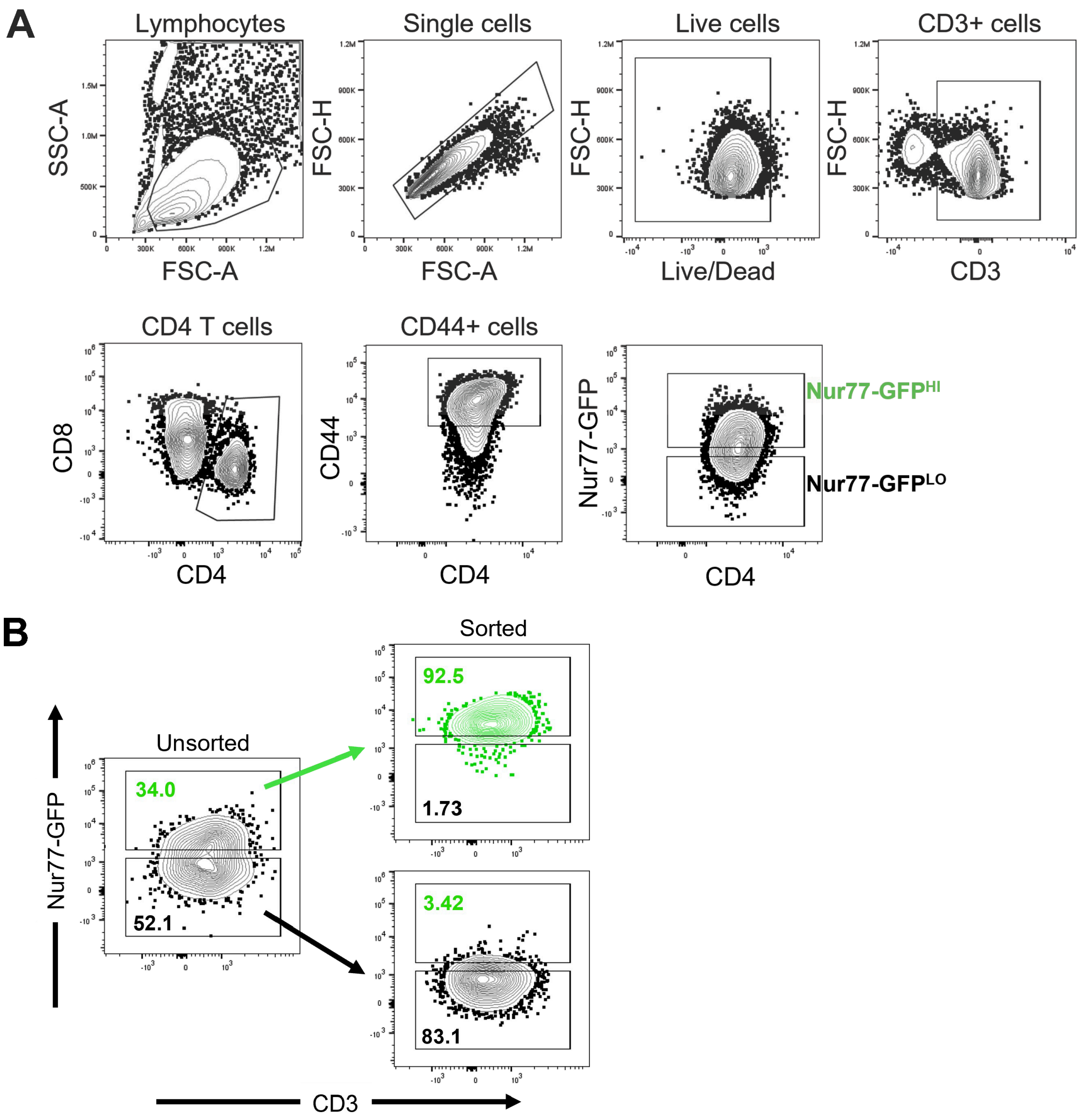

Supplementary figure S2. Nur77-GFP^{HI} and Nur77-GFP^{LO} CD4 T cell sorting strategy and purity.

(**A**) Representative flow cytometry of the sorting scheme for Nur77-GFP^{LO} and Nur77-GFP^{HI} CD4 T cells isolated from Nur77-GFP lungs at four weeks post-infection. Cells gated on lymphocytes, single cells, live/dead stain^{-} for live cells, CD3^{+} for T cells, CD4^{+}CD8^{-} for CD4 T cells, CD44^{+} for effector CD4 T cells, and Nur77-GFP^{LO} and Nur77-GFP^{HI} cells. **(B)** Representative flow cytometry of Nur77-GFP lungs at four weeks post-infection before sorting and post-sort purity of separate Nur77-GFP^{LO} and Nur77-GFP^{HI} CD4 T cell samples. Samples sorted on live CD3^{+}CD4^{+}CD44^{+} Nur77-GFP^{LO} or Nur77-GFP^{HI} cells. Flow cytometry gated on live CD3^{+}CD4^{+}CD44^{+} bulk, Nur77-GFP^{LO}, or Nur77-GFP^{HI} cells.

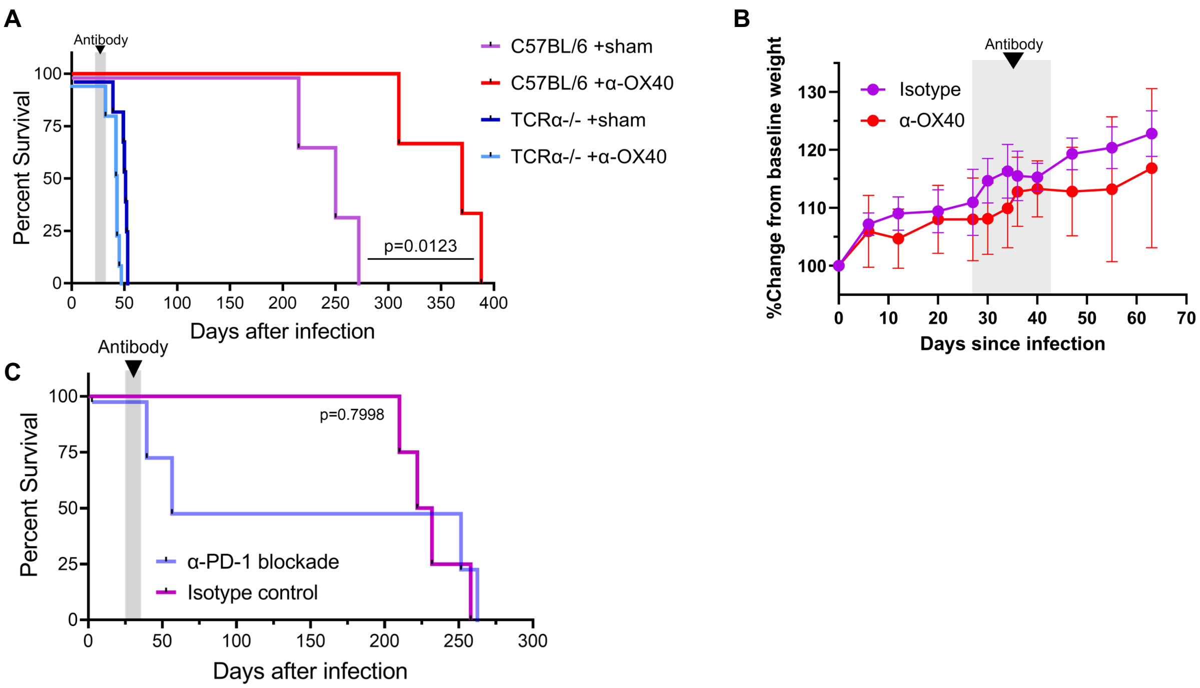

Supplementary figure S3. OX40 agonist treatment improves T cell dependent survival and does not cause adverse effects, in contrast to PD-1 blockade.
(A) Wild type C57BL/6 or TCRα-/- mice at four weeks post-infection treated with 100 µg OX40 agonist antibody or PBS sham injections twice weekly for two weeks. Mice followed for survival. P-value calculated with a two sided Mantel-Cox test. Mice per group: 6 each for both TCRα-/- groups, 3 each for both C57BL/6 groups. **(B)** Wild type mice treated with 100 µg OX40 agonist or isotype control antibody injections twice weekly for two weeks. Mice weighed weekly and percent change from baseline weight at day 0 post-infection calculated for each timepoint. 10 mice per treatment group. Error bars indicate standard deviation. (**C**) Wild type mice were treated at four weeks post-infection treated with 100 µg PD-1 blocking antibody or isotype control injections twice weekly for two weeks. Mice followed for survival. P-value calculated with a two sided Mantel-Cox test with 4 mice per group.

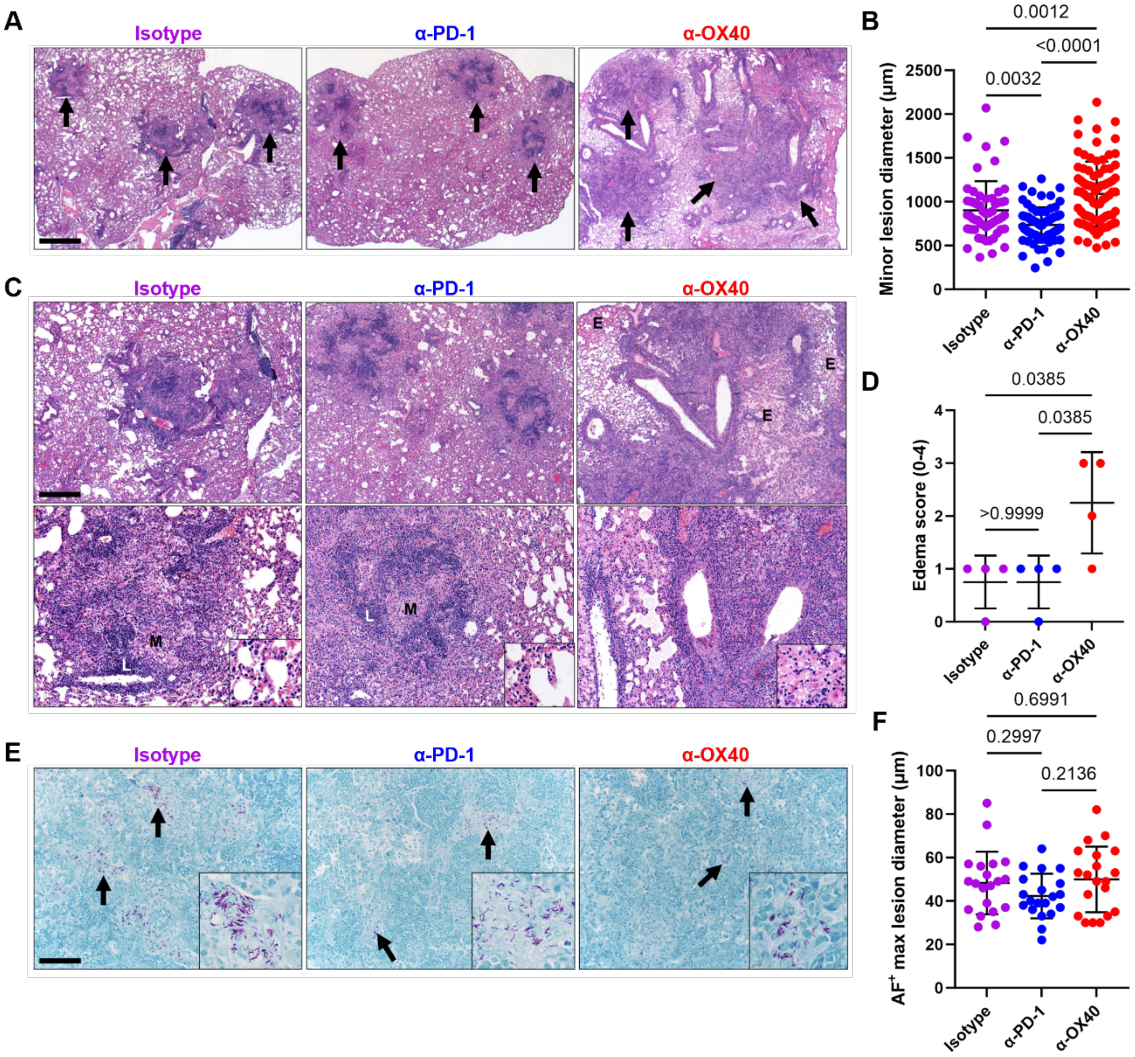

Supplementary Figure S4. OX40 agonism alters lung inflammation.

(A-D) Lesion analysis of antibody-treated lungs. Wildtype C57BL/6 mice at four weeks post-infection treated with 100 μg α-OX40 agonist, PD-1 blockade, or isotype control antibody injections twice weekly for two weeks. Lungs harvested one day after the last antibody treatment for histopathology. Images representative of 4 mice per group. **(A)** HE staining. Cellular inflammation indicated by arrows. Reference bar is 864 μm. **(B)** Minor axis diameter of inflammatory aggregates assessed by a blinded comparative veterinary pathologist. P-values calculated with a one-way ANOVA with post-hoc testing, 4 mice per group. **(C)** HE staining. Cell aggregates labeled with M (macrophages) or L (lymphoid). Areas of edema labeled with E. Reference bar is 432 μm and 174 μm for top and bottom images, respectively. **(D)** Extent of edema ordinarily scored by a blinded comparative veterinary pathologist. P-values calculated with a one-way ANOVA with post-hoc testing, 4 mice per group. **(E)** AFB staining (red color). AFB+ macrophage aggregates are indicated by arrows and insets. Reference bar is 86 μm. **(F)** AF^+ lesion diameter analyzed for the 5 largest lesions in each mouse. P-values calculated with a one-way ANOVA with post-hoc testing, 4 mice per group. Error bars indicate standard deviation.

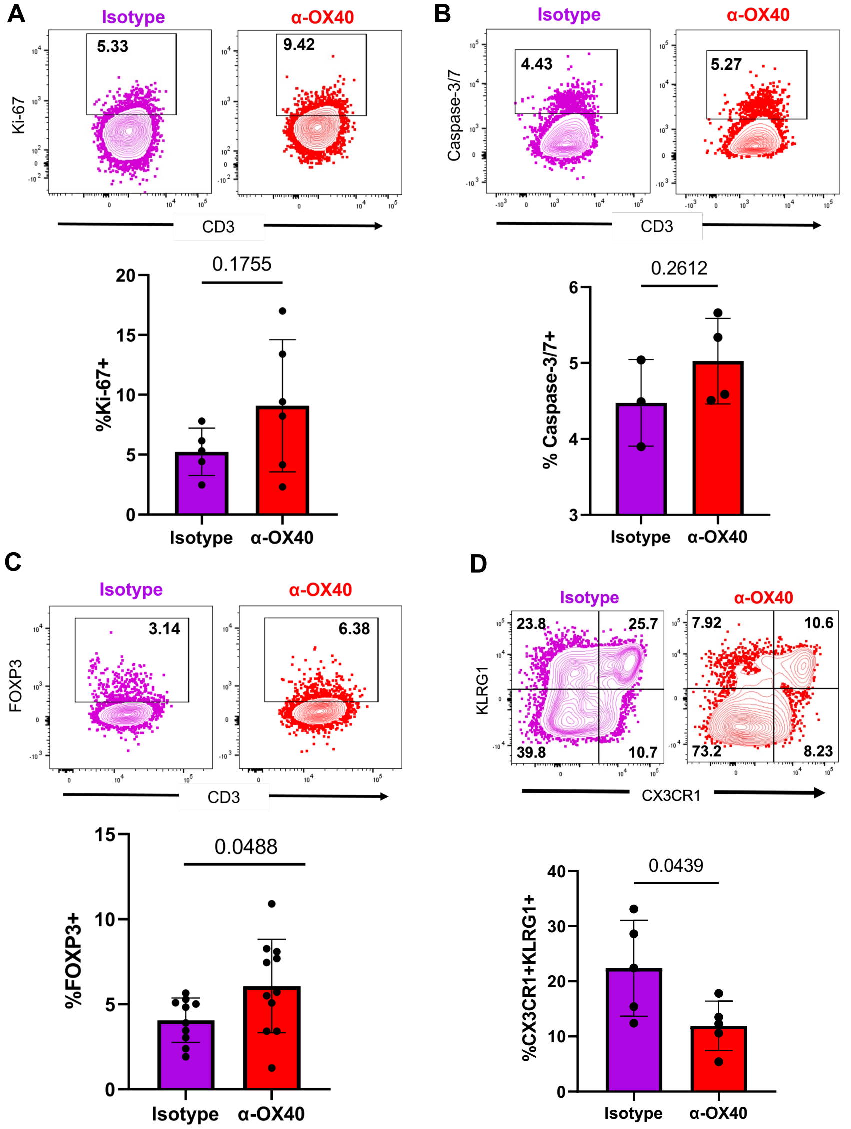

Supplementary figure S5. OX40 agonism shifts CD4 T cell phenotypes without significantly impacting the fraction of proliferating or apoptotic cells.
(A-C) Wild type mice at four weeks post-infection treated with 100 µg OX40 agonist or isotype control antibody injections twice weekly for two weeks. Lungs harvested immediately after treatment course completion for flow cytometry and determination of **(A)** frequency of intracellular Ki-67 expression, 5 mice per group for isotype, 6 mice for OX40. **(B)** Caspase-3/7 expression, 3 mice per group for isotype, 4 mice per group for OX40 **(C)** FOXP3 expression among effector CD4 T cells. Gated on $CD3^{+}CD4^{+}CD44^{+}$ T cells. 10 mice per group for isotype, 10 mice per group for OX40. (**D**) Among Ag85B and ESAT-6 tetramer positive cells, the expression of terminal differentiation markers KLRG1 and CX3CR1, gated on $CD3^{+}CD4^{+}CD44^{+}$tetramer+ T cells. P-values calculated with an unpaired two tailed t-test. 5 mice per group. Error bars indicate standard deviation.

Antibodies	Supplier	Catalog	Clone	Dilution
TotalSeq C0157 CD45.2 (BioLegend 109855)	Biolegend	109855	104	1:100
BUV395 CD4 (BD Biosciences 565974)	BD Biosciences	565974	GK1.5	1:200
BUV496 CD4 (BD Biosciences 612952)	BD Biosciences	612952	GK1.5	1:200
BV421 CD4 (BD Biosciences 562891)	BD Biosciences	562891	GK1.5	1:200
BV785 CD4 (BioLegend 100551)	Biolegend	100551	RM4-5	1:200
redFluor710 CD4 (Cytek 80-0041-U025)	Cytek	80-0041-U025	GK1.5	1:200
BV650 CD3 (BioLegend 100229)	Biolegend	100229	17A2	1:100
BV711 CD3 (BioLegend 100241)	Biolegend	100241	17A2	1:100
PE-CF594 CD3e (BD Biosciences 562332)	BD Biosciences	562332	145-2C11	1:100
BUV737 CD8a (BD Biosciences 612759)	BD Biosciences	612759	53-6.7	1:200
PerCP-Cy5.5 CD8a (Cytek 65-0081-U025)	Cytek	65-0081-U02[illegible]	53-6.7	1:200
APC-eFluor780 CD11b (ThermoFisher 47-0112-82)	ThermoFisher	47-0112-82	M1/70	1:200
PerCPCy5.5 CD11b (Cytek 65-0112-U025)	Cytek	65-0112-U025	M1/70	1:200
APC-eFluor780 CD19 (ThermoFisher 47-0193-82)	ThermoFisher	47-0193-82	1D3	1:200
PerCPCy5.5 CD19 (ThermoFisher 45-0199-42)	ThermoFisher	45-0193-82	1D3	1:200
PE/Cy7 CD44 (BioLegend 103029)	Biolegend	103029	IM7	1:100
BV785 CD44 (BioLegend 103041)	Biolegend	103041	IM7	1:100
APC-Cy7 CD45.2 (Cytek 25-0454-U025)	Cytek	25-0454-U025	104	1:200
redFluor710 CD45.2 (Cytek 80-0454-U025)	Cytek	80-0454-U025	104	1:200
APC CD45.1 (Cytek 20-0453-U025)	Cytek	20-0453-U025	A20	1:200
BV785 CX3CR1 (BioLegend 149029)	Biolegend	149029	SA011F11	1:100
BV605 CD183 (CXCR3) (BioLegend 126523)	Biolegend	126523	CXCR3-173	1:100
BV510 KLRG1 (MAFA) (BioLegend 138429)	Biolegend	138429	2F1/KLRG1	1:100
PE/Dazzle594 CD134 (OX-40) (BioLegend 119417)	Biolegend	119417	OX-86	1:100
BUV395 Ki-67 (BD Biosciences 564071)	BD Biosciences	564071	B56	1:50
PE FOXP3 (BioLegend 126403)	Biolegend	126403	MF-14	1:50
hCD19 BV510 (HIB19) (BioLegend 302241)	Biolegend	302241	HIB19	1:100
hCD4 BV605 (OKT4) (BioLegend 317437)	Biolegend	317437	OKT4	1:100
hCD8 APC-Cy7 (BD Biosciences 557834)	BD Biosciences	557834	SK1	1:100
hCD3 AF488 (Biolegend 300415)	Biolegend	300415	UCHT1	1:100
hCD134 PE (Biolegend 350004)	Biolegend	350004	BER-ACT35	1:100

Non-antibody flow cytometry reagents				
Fixable Viability Dye eFluor780 (ThermoFisher 65-0865-14)	ThermoFisher	65-0865-14	N/A	1:1000
LIVE/DEAD Fixable Aqua Dead Cell Stain (ThermoFisher L34957)	ThermoFisher	L34957	N/A	1:1000
CellEvent Caspase-3/7 (ThermoFisher C10427)	ThermoFisher	C10427	N/A	1:100

Supplementary Table S1. List of antibody and non-antibody reagents for flow cytometry staining.
