# Supplementary material for: Recently activated CD4 T cells in tuberculosis express OX40 as a target for host-directed immunotherapy
Source: Nat Commun. 2023 Dec 19;14:8423. doi: 10.1038/s41467-023-44152-8 (PMC10728168; doi:10.1038/s41467-023-44152-8)
Supplement: Supplementary file 3 — Description of Additional Supplementary Files [file 41467_2023_44152_MOESM3_ESM.pdf]

Description of Additional Supplementary Files

Supplementary Data 1: Bulk RNA-Sequencing analysis of Nur77-GFPHI vs. LO CD4 T cells. CD44+ CD4+ T cells in the lungs of four *Mtb* infected Nur77-GFP mice at day 28 post-infection were live sorted from the highest and lowest 1/3 by Nur77-GFP expression using flow cytometry associated cell sorting. RNA extraction was performed followed by bulk RNA-Seq and differential gene expression analysis using DESEQ2. Volcano plot and heatmap shown in main Figure 2A-B. Statistical comparison performed using two-tailed Wald test with Benjamini-Hochberg correction. Genes are ranked by p value adjusted for multiple hypotheses testing with log2 fold change comparing expression in Nur77-GFPHI cells compared to LO.

Supplementary Data 2: Single cell RNA-Sequencing analysis comparing differentially expressed genes among clusters tetramer enriched CD4 T cells. Cells from the lungs of four *Mtb* infected Nur77-GFP mice at day 28 post-infection were magnetically enriched for ESAT-6 and Ag85B tetramer binding cells, then subjected to single cell RNA-Seq (10x Genomics). Clustering of CD4 T cells by transcriptional phenotype and differential gene expression analysis for each cluster compared to all other clusters was performed using Seurat with FindAllMarkers. UMAP shown in main Figure 2C-D. Genes are sorted by adjusted p value using the standard Wilcoxon Rank Sum two sided test by cluster.

Supplementary Data 3: Single cell RNA-Sequencing analysis of Nur77-GFPHI vs. LO CD4 T cells. CD44+ CD4+ T cells in the lungs of a representative *Mtb* infected Nur77-GFP mouse at day 28 post-infection were live sorted from the highest and lowest 1/3 by Nur77-GFP expression using flow cytometry associated cell sorting, then subjected to single cell RNA-Seq (10x Genomics) in two separate reactions. Clustering of CD4 T cells from the Nur77-GFP-HI and LO populations, in separate tabs, by transcriptional phenotype and differential gene expression analysis for each cluster compared to all other clusters was performed using Seurat with FindAllMarkers. UMAPs shown in main Figure 3A. Genes are sorted by adjusted p value using the standard Wilcoxon Rank Sum two sided test by cluster.

Supplementary Data 4: Single cell RNA-Sequencing analysis of Nur77-GFPHI vs. LO CD4 T cells. CD44+ CD4+ T cells in the lungs of four *Mtb* infected Nur77-GFP mouse at day 28 post-infection were live sorted from the highest and lowest 1/3 by Nur77-GFP expression using flow cytometry associated cell sorting, then subjected to single cell RNA-Seq (10x Genomics) in two separate reactions. Percentage of cells from each of these 8 populations expressing genes of interest are shown. Paired two-tailed t test analysis of percent cells expressing each gene, q value adjusted for multiple hypothesis testing corresponding to column dot plot shown in main Figure 3D.

Supplementary Data 5: Single cell RNA-Sequencing analysis of CSF T cells in human HIV-associated TB meningitis. Live, CD4+ and CD8+ T cells from a cryopreserved CSF cell pellet of a subject with culture-positive TB meningitis were sorted using flow cytometry associated cell sorting, then subjected to single cell RNA-Seq (10x Genomics). Clustering of T cells by transcriptional phenotype and differential gene expression analysis for each cluster compared to all other clusters was performed using Seurat with FindAllMarkers. Genes are sorted by adjusted p value using the standard Wilcoxon Rank Sum two sided test by cluster.
