# Supplementary material for: Recently activated CD4 T cells in tuberculosis express OX40 as a target for host-directed immunotherapy
Source: Nat Commun. 2023 Dec 19;14:8423. doi: 10.1038/s41467-023-44152-8 (PMC10728168; doi:10.1038/s41467-023-44152-8)
Supplement: Supplementary file 9 — Reporting Summary [file 41467_2023_44152_MOESM9_ESM.pdf]

nature portfolio

Corresponding author(s): Tyler Bold

Last updated by author(s): Nov 7, 2023

Reporting Summary

Nature Portfolio wishes to improve the reproducibility of the work that we publish. This form provides structure for consistency and transparency in reporting. For further information on Nature Portfolio policies, see our Editorial Policies and the Editorial Policy Checklist.

Statistics

For all statistical analyses, confirm that the following items are present in the figure legend, table legend, main text, or Methods section.

n/a	Confirmed	
☐	☒	The exact sample size (*n*) for each experimental group/condition, given as a discrete number and unit of measurement
☒	☐	A statement on whether measurements were taken from distinct samples or whether the same sample was measured repeatedly
☐	☒	The statistical test(s) used AND whether they are one- or two-sided<br>*Only common tests should be described solely by name; describe more complex techniques in the Methods section.*
☒	☐	A description of all covariates tested
☐	☒	A description of any assumptions or corrections, such as tests of normality and adjustment for multiple comparisons
☐	☒	A full description of the statistical parameters including central tendency (e.g. means) or other basic estimates (e.g. regression coefficient) AND variation (e.g. standard deviation) or associated estimates of uncertainty (e.g. confidence intervals)
☐	☒	For null hypothesis testing, the test statistic (e.g. *F*, *t*, *r*) with confidence intervals, effect sizes, degrees of freedom and *P* value noted<br>*Give P values as exact values whenever suitable.*
☒	☐	For Bayesian analysis, information on the choice of priors and Markov chain Monte Carlo settings
☒	☐	For hierarchical and complex designs, identification of the appropriate level for tests and full reporting of outcomes
☒	☐	Estimates of effect sizes (e.g. Cohen's *d*, Pearson's *r*), indicating how they were calculated

Our web collection on statistics for biologists contains articles on many of the points above.

Software and code

Policy information about availability of computer code

Data collection	None
Data analysis	Alignment, quantification, and normalization of RNA-sequencing reads was performed using DESEQ2 v1.43.0 for bulk RNA-Seq, and 10X Cellranger v7.0.0 for single cell RNA-Seq. Analysis of single cell RNA-Seq data was performed using Seurat v 4.0.6 and scRepertoire v1.10.1 packages. The code used to process and analyze single cell RNA-Sequencing experiments has been posted at https://github.com/tylerbold, assigned DOI: 10.5281/zenodo.10076323.

For manuscripts utilizing custom algorithms or software that are central to the research but not yet described in published literature, software must be made available to editors and reviewers. We strongly encourage code deposition in a community repository (e.g. GitHub). See the Nature Portfolio guidelines for submitting code & software for further information.

Data

Policy information about availability of data

All manuscripts must include a data availability statement. This statement should provide the following information, where applicable:

- Accession codes, unique identifiers, or web links for publicly available datasets
- A description of any restrictions on data availability
- For clinical datasets or third party data, please ensure that the statement adheres to our policy

Raw and processed sequencing data are available at GEO accession number GSE235800. Ugandan HIV pro-viral genome used for HIV read alignment is located at GenBank: AB098330.1.

Research involving human participants, their data, or biological material

Policy information about studies with human participants or human data. See also policy information about sex, gender (identity/presentation), and sexual orientation and race, ethnicity and racism.

Reporting on sex and gender	Sex and gender were not explicitly considered during the design of this study. The findings are not expected to apply exclusively to any particular gender or biological sex. Specimens from 6 subjects were included in this study, of which 1 identified as male and 5 as female.
Reporting on race, ethnicity, or other socially relevant groupings	The human biospecimens included in this study were from individuals of Ugandan residence.
Population characteristics	Specimens from 6 Ugandan individuals are included in this study. Relevant population characteristics include ages ranging from 22-45, and the presence of HIV co-infection, in addition to TB meningitis as a primary diagnosis.
Recruitment	Baseline (pre-treatment) CSF specimens which were collected under a meningitis screening study protocol for research, for which informed consent is provided by participants: "Improving Diagnostics and Neurocognitive Outcomes in HIV/AIDS-related Meningitis." UMN IRB: 0308M51329,. Mulago Hospital IRB: MHREC 1246
Ethics oversight	Studies involving specimens from human subjects were approved by the University of Minnesota Institutional Review Board as well as Mulago Hospital IRB as above.

Note that full information on the approval of the study protocol must also be provided in the manuscript.

Field-specific reporting

Please select the one below that is the best fit for your research. If you are not sure, read the appropriate sections before making your selection.

☒ Life sciences ☐ Behavioural & social sciences ☐ Ecological, evolutionary & environmental sciences

For a reference copy of the document with all sections, see nature.com/documents/nr-reporting-summary-flat.pdf

Life sciences study design

All studies must disclose on these points even when the disclosure is negative.

Sample size	Sample size was determined based on preliminary data and published results from other groups indicating that 4-5 mice per group are required to establish a statistically significant difference of at least 2 fold for flow cytometry parameters, given the intrinsic intra-group variation between mice. In some experiments, we have used larger sample sizes of up 10 mice per group to provide a more accurate assessment of population means. Relevant citations from this field using groups of this sample size include PMIDs: 30202016, 33711270, 34525366.
Data exclusions	None.
Replication	All findings presented in this paper were replicated with at least 2 independent experiments. In some cases multiple experiments were pooled to increase the n for comparison across treatment groups.
Randomization	No randomization was necessary due to the use of inbred strains of mice in equal numbers of males and females, co-housed under the same conditions.
Blinding	For survival, CFU, and histopathology comparisons investigators were blinded to which treatment (OX40 agonist, PD-1 blockade, isotype control) and which donor cell population (Nur77-GFPHI or LO) was administered to each group. Blinding to experimental grouping was not possible for all experimental comparisons in flow cytometry experiments given the need to pre-gate and classify cells as either Nur77-GFPHi or Lo.

Reporting for specific materials, systems and methods

We require information from authors about some types of materials, experimental systems and methods used in many studies. Here, indicate whether each material, system or method listed is relevant to your study. If you are not sure if a list item applies to your research, read the appropriate section before selecting a response.

Materials & experimental systems

n/a	Involved in the study
☐	☒ Antibodies
☒	☐ Eukaryotic cell lines
☒	☐ Palaeontology and archaeology
☐	☒ Animals and other organisms
☒	☐ Clinical data
☒	☐ Dual use research of concern
☒	☐ Plants

Methods

n/a	Involved in the study
☒	☐ ChIP-seq
☐	☒ Flow cytometry
☒	☐ MRI-based neuroimaging

Antibodies

Antibodies used

Antibodies S
TotalSeq C0157 CD45.2 (BioLegend 109855) Biolegend 109855 104
BUV395 CD4 (BD Biosciences 565974) BD Biosciences 565974 GK1.5
BUV496 CD4 (BD Biosciences 612952) BD Biosciences 612952 GK1.5
BV421 CD4 (BD Biosciences 562891) BD Biosciences 562891 GK1.5
BV785 CD4 (BioLegend 100551) Biolegend 100551 RM4-5
redFluor710 CD4 (Cytek 80-0041-U025) Cytek 80-0041-U025 GK1.5
BV650 CD3 (BioLegend 100229) Biolegend 100229 17A2
BV711 CD3 (BioLegend 100241) Biolegend 100241 17A2
PE-CF594 CD3e (BD Biosciences 562332) BD Biosciences 562332 145-2C11
BUV737 CD8a (BD Biosciences 612759) BD Biosciences 612759 53-6.7
PerCP-Cy5.5 CD8a (Cytek 65-0081-U025) Cytek 65-0081-U025 53-6.7
APC-eFluor780 CD11b (ThermoFisher 47-0112-82) ThermoFisher 47-0112-82 M1/70
PerCPCy5.5 CD11b (Cytek 65-0112-U025) Cytek 65-0112-U025 M1/70
APC-eFluor780 CD19 (ThermoFisher 47-0193-82) ThermoFisher 47-0193-82 1D3
PerCPCy5.5 CD19 (ThermoFisher 45-0199-42) ThermoFisher 45-0193-82 1D3
PE/Cy7 CD44 (BioLegend 103029) Biolegend 103029 IM7
BV785 CD44 (BioLegend 103041) Biolegend 103041 IM7
APC-Cy7 CD45.2 (Cytek 25-0454-U025) Cytek 25-0454-U025 104
redFluor710 CD45.2 (Cytek 80-0454-U025) Cytek 80-0454-U025 104
APC CD45.1 (Cytek 20-0453-U025) Cytek 20-0453-U025 A20
BV785 CX3CR1 (BioLegend 149029) Biolegend 149029 SA011F11
BV605 CD183 (CXCR3) (BioLegend 126523) Biolegend 126523   CXCR3-173
BV510 KLRG1 (MAFA) (BioLegend 138429) Biolegend 138429   2F1/KLRG1
PE/Dazzle594 CD134 (OX-40) (BioLegend 119417) Biolegend 119417 OX-86
BUV395 Ki-67 (BD Biosciences 564071) BD Biosciences 564071 B56
PE FOXP3 (BioLegend 126403) Biolegend 126403 MF-14
hCD19 BV510 (HIB19) (BioLegend 302241) Biolegend 302241 HIB19
hCD4 BV605 (OKT4) (BioLegend 317437) Biolegend 317437 OKT4
hCD8 APC-Cy7 (BD Biosciences 557834) BD Biosciences 557834 SK1
hCD3 AF488 (Biolegend 300415) Biolegend 300415 UCHT1
hCD134 PE (Biolegend 350004) Biolegend 350004 BER-ACT35

Non-antibody flow cytometry reagents
Fixable Viability Dye eFluor780 (ThermoFisher 65-0865-14) ThermoFisher 65-0865-14 N/A
LIVE/DEAD Fixable Aqua Dead Cell Stain (ThermoFisher L34957) ThermoFisher L34957 N/A
CellEvent Caspase-3/7 (ThermoFisher C10427) ThermoFisher C10427 N/A

Validation

Validation for each primary antibody was based on available catalog information (example flow cytometry plots from relevant cell types) from each vendor listed above.

Antibodies
TotalSeq C0157 CD45.2 (BioLegend 109855) Biolegend 109855 104 1:100 Each lot of this antibody is quality control tested by immunofluorescent staining with flow cytometric analysis and the oligomer sequence is confirmed by sequencing.

BUV395 CD4 (BD Biosciences 565974) BD Biosciences 565974 GK1.5 1:200 Two color flow cytometric analysis of CD4 expression on mouse splenocytes.   Mouse splenic leucocytes were preincubated with Purified Rat Anti-Mouse CD16/CD32 antibody (Mouse BD Fc Block™) (Cat. No. 553141/553142). The cells were then stained with APC Hamster Anti-Mouse CD3e (Cat. No. 553066/561826) and BD Horizon™ BUV395 Rat Anti-Mouse CD4 (Cat. No. 563790/565794) antibodies. The two-color fluorescence dot plot shows the correlated expression patterns of CD4 versus CD3e for gated events with the forward and side light-scatter characteristic of viable splenic leucocytes. Flow cytometric analysis was performed using a BD™ LSR II Flow Cytometer System.

BUV496 CD4 (BD Biosciences 612952) BD Biosciences 612952 GK1.5 1:200 Two color flow cytometric analysis of CD4 expression on mouse splenocytes. Mouse splenic leucocytes were preincubated with Purified Rat Anti-Mouse CD16/CD32 antibody (Mouse BD Fc Block™) (Cat. No. 553141/553142). The cells were then stained with APC Hamster Anti-Mouse CD3e antibody (Cat. No. 553066/561826) and either BD Horizon™ BUV496 Rat IgG2b, κ Isotype Control (Cat. No. 612954) or BD Horizon BUV496 Rat Anti-Mouse CD4 antibody (Cat. No. 612952) at 0.5 μg/test. The two-color pseudocolor density plot showing the correlated expression of CD4 (or Ig Isotype control staining) versus CD3e was derived from gated events with the forward and side light-scatter characteristic of viable splenic leucocytes. Flow cytometry and data analysis were performed using a BD LSRFortessa™ Cell Analyzer System and FlowJo™ software. Data shown on this Technical Data Sheet are not lot specific.

BV421 CD4 (BD Biosciences 562891) BD Biosciences 562891 GK1.5 1:200 Multicolor flow cytometric analysis of CD4 expression on mouse splenocytes. Splenic leucocytes were stained simultaneously with PE Hamster Anti-Mouse CD3e antibody (Cat. No. 553064/553063/561824) and with either BD Horizon™ BV421 Rat IgG2a, κ Isotype Control (Cat. No. 562603) or BD Horizon™ BV421 Rat Anti-Mouse CD4 antibody (Cat. No. 562891). Two-color flow cytometric dot plots show the correlated expression patterns of CD4 (or Ig Isotype control staining) versus CD3 for gated events with the forward and side light-scatter characteristics of viable splenic leucocytes. Flow cytometry was performed using a BD™ LSR II Flow Cytometer System

BV785 CD4 (BioLegend 100551) Biolegend 100551 RM4-5 1:200 C57BL/6 mouse splenocytes were stained with CD3 PE and CD4 (clone RM4-5) Brilliant Violet 785™.

redFluor710 CD4 (Cytek 80-0041-U025) Cytek 80-0041-U025 GK1.5 1:200 C57Bl/6 splenocytes were stained with 0.06 ug redFluor™ 710 Anti-Mouse CD4

BV650 CD3 (BioLegend 100229) Biolegend 100229 17A2 1:100 C57BL/6 mouse splenocytes were stained with CD19 APC and CD3 (clone 17A2) Brilliant Violet 650™

BV711 CD3 (BioLegend 100241) Biolegend 100241 17A2 1:100 C57BL/6 splenocytes were stained with CD19 APC and CD3 (clone 17A2) Brilliant Violet 711™ (top) or rat IgG2b, κ Brilliant Violet 711™ isotype control (bottom)

PE-CF594 CD3e (BD Biosciences 562332) BD Biosciences 562332 145-2C11 1:100 Two-color flow cytometric analysis of CD3e expressed on mouse splenocytes. BALB/c splenocytes were stained with APC Rat Anti-Mouse CD4 (Cat. No. 553051) and APC Rat Anti-Mouse CD8a (Cat. No. 561093/553035) and either BD Horizon™ PE-CF594 Armenian Hamster IgG1, κ Isotype Control (Cat. No. 562307) or BD Horizon™ PE-CF594 Hamster Anti-Mouse CD3e antibody (562286/562332). The two-color flow cytometric dot plots showing CD3 (or Ig Isotype Control staining) versus CD4 and CD8 were derived from events with the forward and side light-scatter characteristics of viable splenocytes. Flow cytometry was performed using a BD™ LSR II Flow Cytometer System

BUV737 CD8a (BD Biosciences 612759) BD Biosciences 612759 53-6.7 1:200 Two-color flow cytometric analysis of CD8a expression on mouse splenocytes. Mouse splenic leucocytes were preincubated with Purified Rat Anti-Mouse CD16/CD32 antibody (Mouse BD Fc Block™) (Cat. No. 553141/553142). The cells were then stained with FITC Hamster Anti-Mouse CD3e antibody (Cat. No.553061/553062/561827) and either BD Horizon™ BUV737 Rat IgG2a, κ Isotype Control (Cat. No. 612760) or BD Horizon BUV737 Rat Anti-Mouse CD8a antibody (Cat. No. 612759) at 0.5 μg/test. The two-color fluorescence contour plot shows the correlated expression of CD8a (or Ig Isotype Control) versus CD3e for gated events with the forward and side light-scatter characteristic of viable splenic leucocytes. Flow cytometry and data analysis were performed using a BD LSRFortessa™ Cell Analyzer System and FlowJo™ software.

PerCP-Cy5.5 CD8a (Cytek 65-0081-U025) Cytek 65-0081-U025 53-6.7 1:200 C57Bl/6 splenocytes were stained with 0.25 ug PerCP-Cy5.5 Anti-Mouse CD8a

APC-eFluor780 CD11b (ThermoFisher 47-0112-82) ThermoFisher 47-0112-82 M1/70 1:200 Staining of C57BL/6 bone marrow cells with 0.06 μg of Rat IgG2b kappa Isotype Control APC-eFluor® 780

PerCPCy5.5 CD11b (Cytek 65-0112-U025) Cytek 65-0112-U025 M1/70 1:200 C57Bl/6 bone marrow cells were stained with 0.25 ug PerCP-

APC-eFluor780 CD19 (ThermoFisher 47-0193-82) ThermoFisher 47-0193-82 1D3 1:200 Staining of BALB/c splenocytes with 0.125 μg of Anti-Mouse CD19 PerCP-Cyanine5-5

PerCPCy5.5 CD19 (ThermoFisher 45-0199-42) ThermoFisher 45-0193-82 1D3 1:200 Staining of BALB/c splenocytes with 0.06 μg of Anti-Mouse CD19 APC-eFluor® 780

PE/Cy7 CD44 (BioLegend 103029) Biolegend 103029 IM7 1:100 C57BL/6 mouse splenocytes stained with IM7 PE/Cyanine7

BV785 CD44 (BioLegend 103041) Biolegend 103041 IM7 1:100 C57BL/6 mouse splenocytes were stained with CD44 (clone IM7) Brilliant Violet 785™.

APC-Cy7 CD45.2 (Cytek 25-0454-U025) Cytek 25-0454-U025 104 1:200 C57Bl/6 splenocytes were stained with 0.5 ug APC-Cy7 Anti-

Mouse CD45.2 (25-0454)

redFluor710 CD45.2 (Cytek 80-0454-U025) Cytek 80-0454-U025 104 1:200 C57Bl/6 splenocytes were stained with 0.25 ug redFluor™ 710 Anti-Mouse CD45.2

APC CD45.1 (Cytek 20-0453-U025) Cytek 20-0453-U025 A20 1:200 C57Bl/6 or SJL splenocytes were stained with 0.5 ug APC Anti-Mouse CD45.1

BV785 CX3CR1 (BioLegend 149029) Biolegend 149029 SA011F11 1:100 C57BL/6 mouse splenocytes were stained with CD11b APC and CX3CR1 (clone SA011F11) Brilliant Violet 785™

BV605 CD183 (CXCR3) (BioLegend 126523) Biolegend 126523 CXCR3-173 1:100 C57BL/6 mouse splenocytes were stained with CD3 PE and CXCR3 (clone CXCR3-173)

BV510 KLRG1 (MAFA) (BioLegend 138429) Biolegend 138429 2F1/KLRG1 1:100 C57BL/6 mouse splenocytes were stained with NK1.1 APC

PE/Dazzle594 CD134 (OX-40) (BioLegend 119417) Biolegend 119417 OX-86 1:100 C57BL/6 mouse splenocytes were stimulated for three days with Con-A plus IL-2 and then stained with CD134 (clone OX-86) PE/Dazzle™ 594

BUV395 Ki-67 (BD Biosciences 564071) BD Biosciences 564071 B56 1:50 Two-color flow cytometric analysis of Ki-67 expression by proliferating MOLT-4 and noncycling human peripheral blood mononuclear cells. Proliferating cells from the human MOLT-4 (T lymphoblastic leukemia, ATCC CRL-1582) cell line and noncycling peripheral blood mononuclear cells (PBMC) were fixed and permeabilized with 70% ice cold ethanol. The cells were washed twice with BD Pharmingen™ Stain Buffer (FBS) (Cat. No. 554656) and stained with BD Horizon™ BUV395 Mouse Anti-Ki-67 antibody (Cat. No. 564071) according to the BD Biosciences support protocol, Flow Cytometry Staining Protocol for Detection of Ki-67. The cells were then counterstained with BD Via-Probe™ [Cat. No. 555815/555816; contains 7-Amino-Actinomycin D (7-AAD)] to stain DNA. Two-color flow cytometric dot plots showing the correlated expression patterns of 7-AAD staining versus Ki-67 were derived from gated events with the forward and side light-scatter characteristics of intact MOLT-4 cells or PBMC. Flow cytometric analysis was performed using a BD™ LSR II Flow Cytometer System.

PE FOXP3 (BioLegend 126403) Biolegend 126403 MF-14 1:50 C57BL/6 splenocytes were surface stained with CD4 APC and then treated with True-Nuclear™ Transcription Factor Buffer Set. Cells were then stained with FOXP3 (clone MF-14) PE

hCD19 BV510 (HIB19) (BioLegend 302241) Biolegend 302241 HIB19 1:100 Human peripheral blood lymphocytes were stained with CD3 APC and CD19 (clone HIB19) Brilliant Violet 510

hCD4 BV605 (OKT4) (BioLegend 317437) Biolegend 317437 OKT4 1:100 Human peripheral lymphocytes were stained with CD4 (clone OKT4) Brilliant Violet 605

hCD8 APC-Cy7 (BD Biosciences 557834) BD Biosciences 557834 SK1 1:100 Human peripheral blood lymphocytes were stained with CD8 APC-Cy7

hCD3 AF488 (Biolegend 300415) Biolegend 300415 UCHT1 1:100 Human peripheral blood lymphocytes stained with UCHT1 Alexa Fluor 488

hCD134 PE (Biolegend 350004) Biolegend 350004 BER-ACT35 1:100 3-day PHA-activated human peripheral blood lymphocytes were stained with OX-40 (clone Ber-ACT35) PE

Non-antibody flow cytometry reagents
Fixable Viability Dye eFluor780 (ThermoFisher 65-0865-14) ThermoFisher 65-0865-14 N/A 1:1000 BALB/c thymocytes were uncultured (left) or cultured overnight at 37°C (right) and then stained with Fixable Viability Dye eFluor™ 780.

LIVE/DEAD Fixable Aqua Dead Cell Stain (ThermoFisher L34957) ThermoFisher L34957 N/A 1:1000 Jurkat cells were heat treated at 60 degrees celsius or left untreated, mixed together and stained with LIVE/DEAD® Fixable Aqua Dead Cell Stain Kit, and then fixed with 3.7% formaldehyde. Cells were analyzed by flowcytometry using 405 nm excitation with 530/30 nm emission filte

CellEvent Caspase-3/7 (ThermoFisher C10427) ThermoFisher C10427 N/A 1:100 Jurkat cells (T-cell leukemia, human) were treated with (A) DMSO or (B) 10 µM camptothecin for 3 hours before labeling with the CellEvent® Caspase 3/7 Green Flow Cytometry kit. Stained samples were analyzed on the Attune® Acoustic Focusing Cytometer equipped with a 488-nm laser, and fluorescence emission was collected using a 530/30 BP filter for CellEvent® Caspase 3/7 Green Detection Reagent and a 690/50BP filter for SYTOX® AADvanced™ stain, respectively.

Animals and other research organisms

Policy information about studies involving animals; ARRIVE guidelines recommended for reporting animal research, and Sex and Gender in Research

Laboratory animals	C57BL/6, TCRa-/-, Nur77-GFP. Mice were infected between the ages of 8-12 weeks and monitored for up to 1 year post-infection. Mice were housed in SPF conditions, using a 50/50 dark/light cycle, with ambient temperature ranging from 67-72 degrees F, and relative humidity of 27-60%.
Wild animals	No wild animals were used in the study.
Reporting on sex	Equal numbers of males and females were used for each mouse experiment. There is no expected sex-specific effect.
Field-collected samples	No field collected samples were used in the study.
Ethics oversight	Approval for mouse studies was received by the University of Minnesota Institutional Animal Care and Use Committee

Note that full information on the approval of the study protocol must also be provided in the manuscript.

Flow Cytometry

Plots

Confirm that:

- [ ] The axis labels state the marker and fluorochrome used (e.g. CD4-FITC).
- [x] The axis scales are clearly visible. Include numbers along axes only for bottom left plot of group (a 'group' is an analysis of identical markers).
- [x] All plots are contour plots with outliers or pseudocolor plots.
- [x] A numerical value for number of cells or percentage (with statistics) is provided.

Methodology

Sample preparation	Mice were euthanized with CO2 and lungs were suspended in a digestion buffer containing DMEM, FBS, Collagenase D, DNase, Heparin, CaCl2, and MgCl2. Lungs were incubated at 37°C for 30 minutes and dissociated with a gentleMACS dissociator (Miltenyi Biotec), according to the manufacturer's protocol. To isolate cells, lung suspensions were filtered through a 70 µm cell strainer.
Instrument	Fortessa H1770 (BD Biosciences) or LSR Fortessa X-20 (BD Biosciences) or MA-900 (Sony)
Software	FACSDiva, FlowJo
Cell population abundance	Precision count beads (BioLegend) were added immediately before flow cytometry to enable cell enumeration
Gating strategy	Cells were gated on FSC/SSC parameters consistent with lymphocytes, then on FSC-H/FSC-W to identify singlets, then using live/dead stain to identify live cells, then, on CD3 to identify T cells, then on CD4+, CD8- cells to identify CD4 T cells, then on CD44hi cells to identify effector cells. Cells for Nur77-GFP analysis were gated on the highest and lowest ~33% of GFP signal.

- [x] Tick this box to confirm that a figure exemplifying the gating strategy is provided in the Supplementary Information.
